# Spectrum of Large- and Medium-Vessel Vasculitis in Adults: Neoplastic, Infectious, Drug-Induced, Autoinflammatory, and Primary Immunodeficiency Diseases

**DOI:** 10.1007/s11926-022-01083-5

**Published:** 2022-08-03

**Authors:** Fabian Lötscher, Roxana Pop, Pascal Seitz, Mike Recher, Luca Seitz

**Affiliations:** 1 Department of Rheumatology and Immunology, Inselspital, University Hospital, University of Bern, Freiburgstrasse, CH-3010 Bern, Switzerland; 2 Department of Infectious Diseases and Hospital Hygiene, University Hospital, University of Zurich, Zurich, Switzerland; 3 Immunodeficiency Laboratory, Department of Biomedicine, University Hospital and University of Basel, Basel, Switzerland; 4 University Center for Immunology, University Hospital, Basel, Switzerland

**Keywords:** Vasculitis, Differential diagnosis, Primary immunodeficiency disease, Autoinflammation, Infectious vasculitis

## Abstract

**Purpose of Review:**

To provide a comprehensive review of drugs and neoplastic, infectious, autoinflammatory, and immunodeficiency diseases causing medium- to large-vessel vasculitis in adults with emphasis on information essential for the initial diagnostic process.

**Recent Findings:**

Entities with medium- to large-vessel vasculitis as clinical manifestations have been described recently (e.g., adenosine deaminase-2 deficiency, VEXAS-Syndrome), and vasculitis in established autoinflammatory or immunodeficiency diseases is increasingly being identified.

**Summary:**

In the diagnostic process of medium- to large-vessel vasculitis in adults, a large variety of rare diseases should be included in the differential diagnosis, especially if diagnosis is made without histologic confirmation and in younger patients. Although these disorders should be considered, they will undoubtedly remain rare in daily practice.

**Supplementary Information:**

The online version contains supplementary material available at 10.1007/s11926-022-01083-5.

## Introduction

Advanced genetic analyses and novel pharmacotherapeutics or microbes led to the identification of new disease entities with vasculitic manifestations during the last two decades, and we expect more to be identified in the future. Thus, an ever-increasing variety of vasculitides and vasculitis mimics should be considered in the diagnostic process of adults with suspected medium- or large-vessel vasculitis (MVV, LVV, respectively). This is especially important in situations where histological confirmation of a vasculitic process is not possible (i.e., vasculitis of aorta, coronaries, etc.) and where the final diagnosis rests entirely on clinical, laboratory, and imaging findings. Also, histological confirmation is now commonly omitted if imaging is compatible with MVV and/or LVV, but in atypical cases or potential mimics, caution is advised [1, 2].

In this article, autoinflammatory, primary immunodeficiency, neoplastic, or infectious diseases as well as drugs causing MVV and/or LVV are comprehensively reviewed. The primary vasculitides and vasculitis in arthritides, connective tissue, and fibroinflammatory diseases as well as vasculitis mimics are reviewed elsewhere [3]. Single-organ vasculitides have been recently reviewed in this journal [4]. The problem of the inconsistent and variable definition of vessel size has been discussed recently [3]. Because Chapel Hill’s nomenclature remains ambiguous, we defined the vessel sizes according to our daily practice, which is similar to Chapel Hill, but attempts to define large vessels more specifically (see legend of Table 1) [3, 5]. Some of the described diseases exceptionally cause MVV or LVV and are mentioned in the text body only. For diseases that are more regularly associated with MVV and LVV and where a larger body of evidence exists, further details important for recognizing the disease and making the initial diagnosis are presented in the tables: Tables 1 and 2 are abridged versions of the Supplementary Tables S1 and S2, where the complete information (including details on clinical manifestations and important laboratory findings) can be found.

**Table 1:** Neoplastic, autoinflammatory, and primary immunodeficiency diseases and drugs causing large- and medium-vessel vasculitis in adults

Category	Disease	Epidemiology, patient characteristics	Mainly affected large and medium vessels or vessel beds (i)		Diagnostic pearls and pitfalls (ii)
			Large	Medium	
Autoinflammatory diseases	Familial Mediterranean fever	FMF: onset childhood to early adulthood, AR inheritance (MEFV gene), prevalence Mediterranean region higher. “PAN-like” MVV: ~ 1% of FMF; m > f = 3.6:1; usually after onset of FMF. BS: associated with FMF in ~ 0.4%, f > m [6–8]	+ / − aorta (thoraco-abdominal); CCA, subclavian (“TAK-like”) [9, 10]	‡ “PAN-like” renal, cerebral, abdominal, cutaneous + / − cardiac [6]	• Perirenal hematoma in ~ 50% (distinctive feature of “PAN-like” MVV) • “BS-FMF-overlap”: cutaneous, gastrointestinal and CNS involvement more frequent than in isolated BS [8] • “PAN-like” MVV: compared to classical PAN, FMF patients are younger, testicular/cardiac involvement less frequent; CNS involvement more frequent and GN is possible • Hepatitis B Infection is detected in up to ~ 7% of “PAN-like” FMF [6]
	Inflammatory bowel diseases	IBD onset usually adolescence to early adulthood (onset at any age possible). IBD (esp. CD) and TAK or “TAK-like” at younger age (~ 20 Y/A) than isolated TAK [11, 12]	‡ “TAK-like” or TAK: aorta, subclavian, vertebral [11, 12]	+ / − “TAK-like” or TAK: renal, mesenteric, cerebral, cutaneous, TA (only with associated GCA) [11, 12]	• IBD preceding vasculitis in most cases in ~ 70% • GCA with IBD rarely relapsing (in contrast to isolated GCA) • IBD usually not active at time of vasculitis onset • Due to limited data, differentiation of concomitant TAK or GCA and IBD vs. “TAK-like” and “GCA-like” disease with IBD not possible
	Chronic recurrent multifocal osteomyelitis	Onset usually in childhood, but possible in adults; f > m; globally. LVV very rare (onset 3 to ~ 50 Y/A) [13, 14]	‡ “TAK-like”: aorta (ascending, descending), CCA, subclavian [13, 14]	(iii)	• Further associations with pyoderma gangrenosum, synovitis, acne, pustulosis, hyperostosis, osteitis syndrome (SAPHO) and IBD [13]
Primary immunodeficiency diseases	Common variable immunodeficiency	May manifest in childhood or in adulthood at any age; m = f; less common in developing nations; true prevalence of CVID unknown, estimated ~ 0.5–7/10^6^; any form of vasculitis in ~ 2% of CVID [15••, 16, 17•]	‡ “TAK-like” or TAK: aorta, innominate, CCA, ICA, axillary, subclavian [18–20]	+ / − “TAK-like” or TAK: renal, mesenteric, celiac, coronary [18–20]	• Rule out other causes of hypogammaglobulinemia before diagnosing CVID • Detection of aortic aneurysm in CVID should trigger imaging for LVV • Consider deficiency of adenosin-deaminase-2 in CVID, esp. in patients with MVV • Screen for splenomegaly and lung disease in potential CVID • Limited significance of any serologic test if patient is receiving immunoglobulin replacement therapy
	Wiskott-Aldrich syndrome	Usually diagnosis in early childhood, exceptionally delayed to early adulthood in milder variants; X-linked recessive disorder; prevalence ~ 4/10^6^; any form of vasculitis in ~ 1 – 29% [21, 22]	‡ aorta (frequent aortic aneurysms, often panaortic); + / − aortic arch arteries (“TAK-like”) [23, 24]	+ / − cerebral, kidney, cardiac, liver, bowel, stomach [25, 26]	• With longer survival of patients with Wiskott-Aldrich, aneurysms secondary to LVV may increasingly become recognized; screening beginning in childhood might be justified • Isolated presentation with thrombocytopenia is commonly called “X-linked thrombocytopenia”, a mild variant of Wiskott-Aldrich • Missing or reduced expression of the “WAS-protein” can be detected rapidly by lymphocyte flow cytometry in peripheral blood [21]
	Deficiency of adenosin-deaminase-2	Variable disease onset, can be delayed to adulthood; AR inheritance; m = f; globally (less common in Africa, East Asia); estimated prevalence ~ 4.5/10^6; any vasculitic feature ~ in > 75–90% [27•, 28, 29•]	(iii)	‡ “PAN-like” (including aneurysms): skin, muscle, mesenteric, celiac, hepatic, renal + / − splenic, testicular, cerebral, coronary, pancreatic, TA [27•, 29•, 30]	• Screening with ADA2 activity testing (e.g., with dried plasma spots) • Biallelic mutations can be found in asymptomatic individuals (usually through screening of seemingly unaffected family members) • Sneddon syndrome is an important differential diagnosis (livedo racemosa and CNS lesions) • CNS involvement is much more common in DADA2 than in PAN • Skin biopsy can show leukocytoclastic SVV and necrotizing MVV
Malignancy—paraneoplasia Myeloid neoplasms	Myelodysplastic syndromes Myeloproliferative neoplasms	MDS/CMML: median onset ~ 70–75 Y/A (range 16–90 Y/A), m > f (MDS), m = f (CMML). MVV/LVV less frequent in polycythemia vera or essential thrombocythemia [31–33]	+ / − aorta, large veins (“BS-like” manifestation) [31, 34, 35]	‡ TA (“GCA-like”) + / − “PAN-like” (renal, hepatic, mesenteric, TA (non-GCA), cerebral, cutaneous) [31, 36–35]	• Consider MDS/MPN in refractory MVV/LVV or with inflammatory dysimmune phenomena [36] • The finding of cytopenia in LVV or MVV should lead to consideration of MDS or MPN as underlying disease process • MDS with GCA has poorer outcome (relapses ↑, steroid dependency) [33]
	Acute and chronic myeloid leukemia	Any age possible, incidence increases with age. AML: often progression of MDS/MPN. MVV/LVV very rare	+ / − “TAK-like”: aorta, CCA/ICA, innominate, subclavian, axillary [37, 38]	+ / − “PAN-like”: lower leg, TA (“GCA-like”) [39–38]	• Basophilia and eosinophilia are common in chronic myeloid leukemia • A differential blood count with visual inspection is advised in the setting of vasculitis with leukocytosis
Lymphoid neoplasms	Hodgkin and non-Hodgkin lymphomas	Any age possible. Hodgkin and non-Hodgkin lymphoma occasionally with LVV/MVV, multiple myeloma only rarely [39]	+ / − aorta, iliac, femoral [37]	‡ cerebral; + / − “PAN-like”: renal, hepatic, mesenteric, infrabrachial, infrapopliteal, coronary; TA (“GCA-like”) [39, 37, 42–44]	• Lymphocyte flow cytometry of peripheral blood frequently shows monoclonality • Intravascular lymphoma is a potential mimic of MVV, especially in the CNS or skin [3]
	Hairy cell leukemia	Mean onset ~ 50 Y/A (any age possible); m > f; MVV very rare [44, 45]	(iii)	‡ “PAN-like”: cutaneous, hepatic, mesenteric, renal, cerebral, TA (occasionally TA aneurysm) [44–46]	• MVV is usually diagnosed in patients with known leukemia [44, 45] • “Hairy” cells can typically be identified in the peripheral blood smear
Other	VEXAS-syndrome	Usually manifests ~ 50–80 Y/A; male > 95%; associated MDS in > 30%; caused by somatic mutation in *UBA1*-gene; subset with polychondritis [47••, 48]	+ / − aorta [47••, 48]	‡ cutaneous (25%) + / − TA [47••, 48]	• Consider VEXAS in refractory cases with inflammatory dysimmune phenomena • Elderly patient with autoinflammatory symptoms, cytopenias, and/or polychondritis: look for vacuoles in bone marrow [47••, 48]
Drug-induced vasculitis	Minocycline	Young patients (average 30 Y/A) with acne treatment (often long-term); f > m. [49, 50]	(iii)	‡ “PAN-like”: skin, nerves + / − renal, mesenteric, gall bladder, liver, spleen, cervix [49, 50]	• Onset of MVV on average ~ 26 months after initiation of minocycline therapy [49, 50]
	Immune checkpoint inhibitors	Onset ~ 40–70 Y/A; f = m; vasculitis typically 1–3 months after initiation of treatment (*ipilimumab, pembrolizumab, nivolumab)* [51, 52]	+ / − aorta [51]	‡ TA (“GCA-like”), cerebral (similar to primary angiitis of the CNS), uterine and ovarian vessels; peripheral nerves [51]	• Vasculitis typically resolves after stopping immunotherapy (and/or a course of oral or intravenous glucocorticoids) • No fatalities related to vasculitis observed [51] • Overlap with other immunotherapy-related adverse events possible (pericarditis, myocarditis, endocrine, gastrointestinal, etc.) [52]
	Granulocyte colony–stimulating factor	Vasculitis occurs in ~ 0.3–0.5% of patients receiving G-CSF; mean ~ 60 Y/A; f > m [53, 54]	‡ aorta (abdominal < thoracic or panaortic); carotids; + / − iliac, femoral, innominate, subclavian [53, 54]	+ / − TA [53]	• No fatalities related to vasculitis were observed • In about 60%, vasculitis occurs within 10 days after G-CSF initiation (agents: pegfilgrastim; filgrastim; lipefilgrastim; lenograstim)
	Graft versus host disease	Vasculitis is a very rare manifestation	+ / − aorta, iliac, femoral, popliteal, subclavian[55]	+ / − cerebral [56]	• Cerebral vasculitis can manifest in long-term survivors [56]

(i) Main vessels or vessel beds identified by our literature searches (i.e., arteries or vessel beds not listed, could still be affected). If not specified otherwise, the vessel names indicate arteries. The vessel sizes are defined as follows: “Large” (the aorta and distributing vessels of the extremities and neck, originating proximal to the elbow, knee, and dura mater), “small” (arterioles, capillaries, venules, and small intraparenchymal arteries and veins), “medium” (remaining vessels, including visceral arteries). (ii) Pearls mostly reflect the personal experience of the authors and are only partially referenced. (iii) Not identified by our literature search. Special characters: ‡, typically affected vessels or vessel beds; + / − , occasionally to rarely affected vessels or vessel beds; ~ , approximately

*AR* autosomal recessive; *BS* Behçet’s syndrome, *CCA* common carotid artery, *CD* Crohn’s disease, *CNS* central nervous system, *CMML* chronic myelomonocytic leukemia, *CVID* common variable immunodeficiency, *DADA2* deficiency of adenosine-deaminase-2, *ESR* erythrocyte sedimentation rate, *FMF* familial Mediterranean fever, *GCA* giant cell arteritis, *G-CSF* granulocyte colony–stimulating factor, *IBD* inflammatory bowel disease, *ICA* internal carotid artery, *LVV* large-vessel vasculitis, *MDS* myelodysplastic syndrome, *MPN* myeloproliferative neoplasm, *MVV* medium-vessel vasculitis, *PAN* polyarteritis nodosa, *TA* temporal artery, *TAK* Takayasu arteritis, *UC* ulcerative colitis, *VEXAS* vacuoles, E1 enzyme, X-linked, autoinflammatory, somatic, *Y/A* years of age

**Table 2:** Infectious diseases causing large- and medium-vessel vasculitis in adults

Pathogen	Epidemiology, patient characteristics	Mainly affected large and medium vessels or vessel beds (i)	
		Large	Medium
Bacteria/Mycobacteria			
Gram-positive bacteria			
Gram-positive cocci (staphylococci, streptococci, enterococci)	Any age; predisposing conditions for medium- or large-vessel vasculitis: any infection with bacteremia (typically endocarditis), atherosclerosis, ICH, IVDA	‡ aorta, femoral, carotid (distal ICA) [101, 102, 104, 105]	‡ cerebral: mainly anterior circulation, splanchnic. [101, 105, 106•]
*Listeria* spp.	Elderly or young children; ICH; pregnancy; eating of contaminated products, more common spring/summer	‡ aorta (abdominal > thoracic) [107, 108]	(ii)
*Mycoplasma* spp.	History of atypical pneumonia; exposure to infected children; in context of outbreak	(ii)	‡ cerebral [106•, 109]
Mycobacteria			
*M. tuberculosis*	Travel (long-term) to or migration from high prevalence countries; close contacts (e.g., family member) with active pulmonary tuberculosis; ICH at risk of reactivation of latent infection	‡ aorta (abdominal > thoracic); distal ICA [104, 110]	+ / − proximal cerebral arteries [106•]
Gram-negative bacteria			
Spirochetes			
*Treponema pallidum* (Syphilis)	Sexual activity; other sexually transmitted infections in the past	‡ ascending aorta + / − aortic arch or descending aorta (rarely sinus or abdominal aorta) [111, 112]	+ / − cerebral: anterior circulation (esp. middle cerebral artery) + / − TA [106•, 111, 113]
*Borrelia* spp.	History of tick bite or erythema migrans (< 50%); frequent outdoor activity in endemic regions	(ii)	‡ cerebral: SVV/MVV (diffuse and mainly leptomeningeal) + / − dural sinus [106•]
*Leptospira* spp.	Travel to warm and tropical regions, outdoor activity and animal contact (farmers, veterinarians, soldiers, canal workers (contact to contaminated soil and water) are at high risk)	‡ aorta [114]	+ / − cerebral (size unclear), dural sinus; coronary [106•, 114]
Other Gram-negative bacteria			
*Salmonella* spp.	Atherosclerosis, diabetes mellitus, ICH, hemoglobinopathies, abnormal intestinal mucosal barrier; eating of contaminated food	‡ aorta (abdominal > thoracic) [115]	(ii)
*Coxiella burnetii*	Travel to or migration from endemic regions; close contact to animals (e.g., cattle); occupational exposure (veterinarian, farmer); ICH; pregnancy	‡ aorta, mostly abdominal; + / − axillary [116, 117]	+ / − TA, “PAN-like” possible (hepatic) [117, 118]
*Brucella* spp.	Occupational exposure (farmer, animal breeder, butcher), travel to or migration from endemic regions, drinking of unpasteurized milk	‡ aorta (abdominal > thoracic), ICA [119, 120]	+ / − cerebral, dural sinus [120]
*Francisella tularensis*	Outdoor activity or outdoor profession (e.g., forester, hunter), exposure to rodents, tick bites, contaminated material	‡ aorta (abdominal) [121]	(ii)
Viruses			
Varicella Zoster virus	Any age, ICH at risk	+ / − ICA, vertebral arteries [106•, 122]	+ / − cerebral [106•, 122]
Herpes Simplex virus	Any age; with or without comorbidities	+ / − ICA [123]	‡ cerebral [123, 124]
Cytomegalovirus	Usually in heavily ICH	(ii)	‡ “PAN-like” (distal extremities, renal, splanchnic) [125, 126]
HIV	Migrants from high-burden countries, risk for STD, IVDA, mostly young or middle-aged patients	‡ aorta, carotid, subclavian, femoral, popliteal [127, 128]	‡ cerebral + / − “PAN-like” (skin, nerve, muscle, renal splanchnic) [106•, 127]
Hepatitis B virus	Migrants from high burden countries (also second-generation: perinatal transmission), IVDA, sex workers	(ii) but highly likely	‡ “PAN-like” (skin, renal, testicular, splanchnic, peripheral nerves) [101, 105, 129, 130]
Fungal infections (yeasts (*Cryptococcus, Candida* spp.), molds (*Aspergillus*, *Mucor* spp.), dimorphic (*Histoplasma*, *Coccidioides* spp.))	ICH, critically ill patients, IVDA; migration from and long-term travel to endemic regions (dimorphic fungi)	+ / − aorta, carotids [131–133]	‡ proximal cerebral arteries (including mycotic aneurysms), esp. Candida and Aspergillus spp. [105, 134, 135]
Parasites (iii)			
*Taenia solium* (neurocysticercosis)	Migration from endemic region (seroprevalence in endemic regions is very high)	+ / − ICA [136]	‡ cerebral (“cysticercal arteritis”) [136]
*Toxocara* spp.	Close contact to dogs/cats (ownership); migration from and travel to endemic regions	(ii)	‡ cerebral (all vessels can be affected) [137]

(i) Main vessels or vessel beds identified by our literature search (i.e., arteries or vessel beds not listed, could still be affected). If not specified otherwise, the vessel names indicate arteries. (ii) Not identified by our literature search. (iii) Only two common parasitic infections are mentioned due to the rarity of such cases in Western Europe. Special characters: ‡, typically affected vessels or vessel beds; + / − , occasionally to rarely affected vessels or vessel beds

*CNS* central nervous system, *ICA* internal carotid artery, *ICH* immunocompromised host (e.g., immunosuppressive therapy, solid organ or hematopoietic stem cell transplantation, HIV), *IVDA* intravenous drug abuse, *PAN* polyarteritis nodosa, *spp* species pluralis, *STD* sexually transmitted diseases

## Autoinflammatory Diseases

Autoinflammatory diseases (AIDs) are characterized by systemic inflammation due to dysregulation of the innate immune system. They can be broadly divided into monogenic and polygenic diseases. Some may present with vasculitis in adults [57••, 58•].

## Monogenic Autoinflammatory Diseases

Most monogenic AIDs manifest in early childhood, but occasionally onset or delayed diagnosis in adulthood are reported. Vasculitis and vasculopathy of the large- and medium-sized vessels are rare manifestations of these diseases [58•].

*Familial Mediterranean fever* (FMF) is the most common monogenic AID, typically presenting with recurrent episodes of fever, abdominal pain, rash, and arthralgia, and is also the most strongly associated with systemic vasculitis: IgA vasculitis occurs in 2.7–7%, and a vasculitis similar to polyarteritis nodosa (PAN) can be observed in approximately 1% [6, 59]. Interestingly, perirenal hematomas are a frequent manifestation and some patients present with glomerulonephritis, which is not observed in classical PAN [6]. An independent, FMF-associated form of vasculitis can be postulated, hence the term “PAN-like” vasculitis has been proposed [6]. LVV such as Takayasu arteritis (TAK) or Cogan Syndrome have rarely been described to co-occur with FMF [6, 9, 10]. Particularly noteworthy is the possible co-occurrence of Behçet’s syndrome (BS) and FMF. Both diseases have a higher prevalence in the Mediterranean region. They share certain clinical manifestations (such as fever, arthritis, cutaneous signs, and the self-limited relapsing disease course), both respond to colchicine, and in cohorts of BS, FMF is more common than in the general population in the same region [7, 8]. *Pyogenic arthritis, pyoderma gangrenosum, and Acne (PAPA) syndrome* is another monogenic AID that can rarely present with vasculitis; a single patient with possible cerebral vasculitis and aneurysm of the posterior cerebral artery was identified [58•, 60]. The *tumor necrosis factor receptor–associated periodic syndrome (TRAPS)*, typically presenting with recurrent episodes of fever, periorbital edema, abdominal pain, arthralgia/myalgia, and rash, is rarely associated with MVV and SVV, but disease onset in adulthood is rare, and so far, vasculitides have primarily been described in children [58•].

The *Aicardi-Goutières syndrome* (AGS) and *STING-associated vasculopathy with onset in infancy* (SAVI) are interferonopathies that almost exclusively manifest in childhood. Rare cases of adult-onset or delayed diagnosis in adulthood with vasculitic manifestations are reported. Clinical manifestations of AGS include encephalopathy, dystonia, spasticity and cognitive impairment, characteristic calcifications of the basal ganglia, and various autoimmune features, ranging from low-titer antinuclear antibodies (ANA) to the full clinical spectrum of systemic lupus erythematosus. Cerebral MVV with arterial stenoses and stroke can very rarely occur in adults, especially in patients with mutations in the *SAMHD1* gene [57••, 61, 62]. SAVI, an autosomal dominant disease, presents with a broad clinical spectrum, such as interstitial lung disease, skin rashes/plaques, arthritis, and MVV, which may occasionally lead to distal limb ischemia with consecutive need for amputation [61–63].

The spectrum of monogenic AIDs is continuously expanding. Two more recently described diseases, *deficiency of adenosine deaminase 2 (DADA-2)* and *Haploinsufficiency A20 (HA20)*, can also present with vasculitis in adults [27•, 64••]. HA20 is an autosomal dominant relopathy that usually begins in childhood with a relapsing–remitting course. Rarely, the diagnosis is delayed to adulthood. While the clinical phenotype is very similar to BS, polyarthritis, fever, and fluctuating antibody positivity (e.g., ANA, anti-ds-DNS antibodies or lupus anticoagulant) are more frequently observed in HA20. MVV of the CNS are occasionally observed, and single cases of pulmonary artery and skin vasculitides have been described. The disease should be sought especially in familial early-onset orogenital and gastrointestinal ulcers with articular and ocular symptoms. [3, 64••, 65]. DADA-2 is described below.

## Polygenic Diseases with Autoinflammatory Features

### Inflammatory bowel disease (IBD)

Crohn’s disease (CD) and ulcerative colitis (UC) have many features of AIDs [66]. Examples of MVV and LVV in patients with IBD are abundant in the literature, but precise epidemiological data are lacking. It has been reported that up to 5% of patients with TAK concomitantly suffer from CD. Gastrointestinal manifestations usually precede vasculitis and LVV is more frequent and often presents with a TAK-pattern [11, 67]. Interestingly, in GCA associated with IBD, male gender seems to be more common [12]. While UC often presents with ANCA (anti-neutrophil cytoplasmic antibodies) and CD with ASCA (anti-*Saccharomyces cerevisiae* antibodies) positivity, the clinical picture of ANCA-associated vasculitis (AAV) is only rarely observed in IBD [68–70]. Suspected genetic (such as shared associations to HLA-B*52 in patients with TAK and UC) and pathophysiological similarities of LVV and IBD (e.g., granulomatous inflammation in both LVV and CD) have been put forward as arguments for a common genesis of the diseases, but data are limited [11]. Future research may tell whether LVV and MVV are features of IBD or the co-occurrence of two different diseases [11, 69]. The overlapping clinical features (intestinal and extraintestinal; e.g., ocular inflammation, erythema nodosum, or oral aphthae) and similar age range may make it difficult to distinguish IBD from BS, another polygenic disorder with autoinflammatory features [71]. Furthermore, Cogan’s syndrome, a variable vessel vasculitis, can rarely be associated with IBD [72]. BS and Cogan’s syndrome are classified as primary vasculitides [3].

In *chronic recurrent multifocal osteomyelitis*, an AID in children with rare onset in adulthood, presenting with bone pain and soft tissue swelling, a “TAK-like” LVV has rarely been described [13, 14].

## Primary Immunodeficiency Diseases

**Fig. 1:**
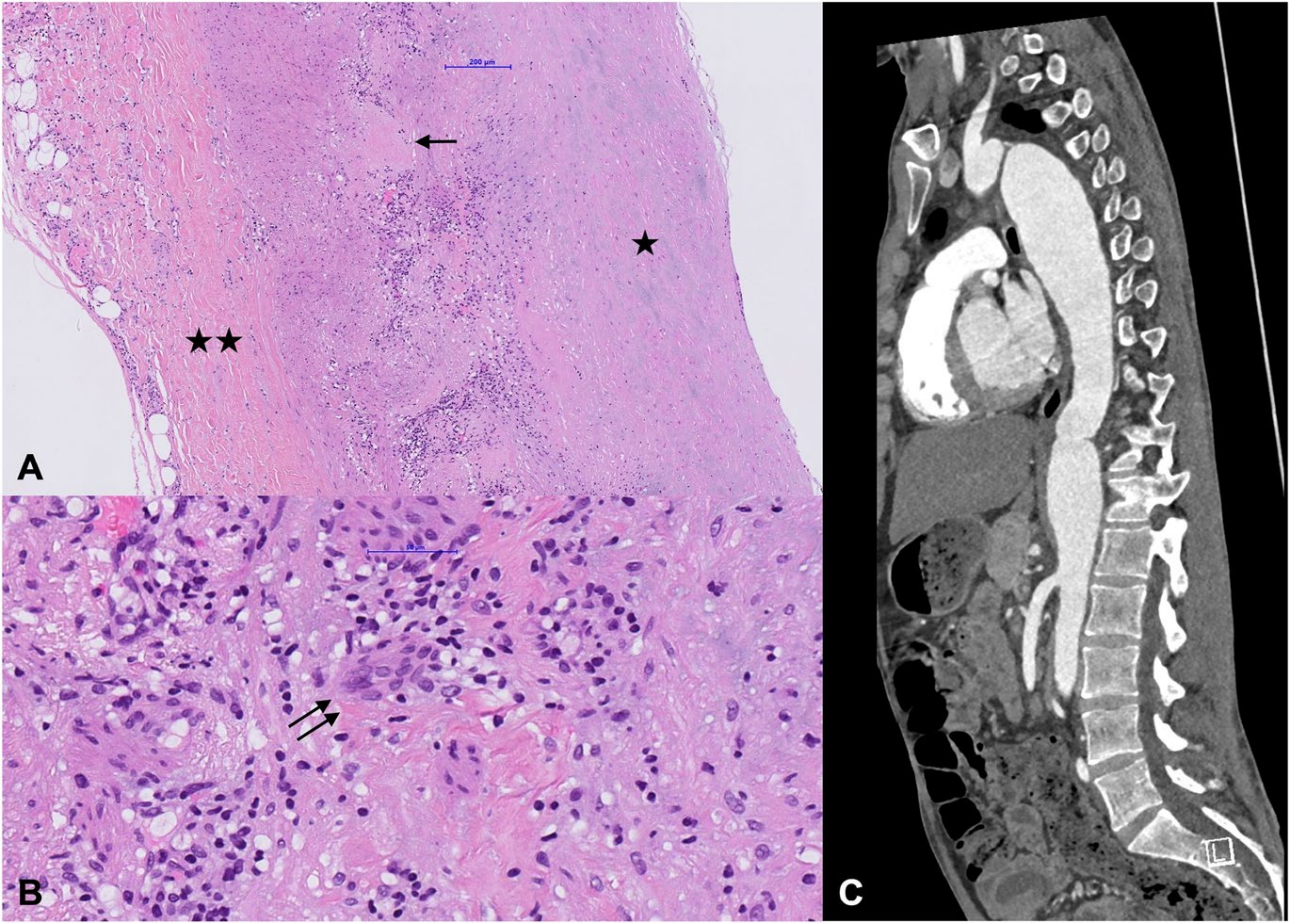
Case description: Male patient with a molecularly unclassified primary immunodeficiency (CVID-like) with frequent major infections in early childhood (e.g., meningitis). Laboratory findings consisted of intermittent neutropenia; mild lymphopenia with normal numbers of total T-cells and NK-cells; reduced naïve T cells, total and class-switched B cells; severely reduced IgG/IgM/IgA. Immunoglobulin replacement therapy was started before the age of 10. He developed a panaortic dilatation already in childhood, and a thoracoabdominal aortic replacement was performed at the age of 23. **A**, **B** Hematoxylin and eosin staining of the descending aorta showing intimal fibrosis (single star), block-like media necrosis (single arrow), transmural lymphohistiocytic infiltration with presence of giant cells (double arrow), and periadventitial fibrosis (double star); extensive microbial analysis of the specimen remained negative. **C** CT angiography of aorta (sagittal view) with aneurysm of the suprarenal abdominal and descending aorta

Primary immunodeficiency (PIDs) are genetically determined diseases which are typically but not always accompanied by susceptibility to infection. The reduced immune tolerance can lead to autoimmune phenomena and/or overt autoimmune diseases. More than 450 PID entities have been identified, and while PIDs are increasingly recognized in adults, they still remain underdiagnosed [73]. Germline mutations of immune genes with only partial alteration of protein function may lead to late-onset manifestations [15••]. In addition, somatic mutations in immune genes may cause phenocopies of PIDs [15••, 73]. In developing countries, access to genetic testing is difficult and undiagnosed PIDs with childhood onset should be considered in migrants, especially from countries with high prevalence of consanguinity. While a presentation with SVV is clearly more common, there are certain PIDs that are associated with MVV and/or LVV (see case study in Fig. 1). The frequent affection of the vasculature of the central nervous system (CNS) in PIDs is noteworthy [25].

### Common variable immunodeficiency (CVID)

CVID is an umbrella diagnosis of patients with suspected PID that present with hypogammaglobulinemia [16, 17•]. CVID in adults is probable, if reduced serum levels of IgG and low IgM or IgA and a poor antibody response to vaccines are discovered in a patient with frequent infections and/or autoimmune features and/or enlarged spleen/lymph nodes. Secondary causes of hypogammaglobulinemia need to be excluded [15••, 16, 17•]. Vasculitis is present in approximately 2% of adults with the diagnosis of CVID [17•]. Because of their rarity, it remains unclear whether the vasculitides in CVID are primary vasculitides in the sense of autoimmune phenomena or whether they are separate entities which may require different therapeutic approaches. All vessel sizes can be involved, including SVV of the CNS and retina, ANCA-positive SVV, or LVV [18–20, 74–76, 77•].

### Wiskott-Aldrich syndrome (WAS)

WAS is a rare X-linked PID and is usually diagnosed in childhood, but vasculitis can present in male adults [21]. Thrombocytopenia with small platelets is the signature finding, and susceptibility to bacterial and viral infections, atopy, and autoimmune features are variably present [21, 78]. Vasculitides are the second most common autoimmune feature (in 1–29% of patients) [22]. While SVV, especially IgA vasculitis, is the most frequent vasculitis, necrotizing MVV can occur in various organs and vessels, including the CNS. In adults, lymphohistiocytic and/or granulomatous LVV with propensity to development of aortic aneurysms, is especially noteworthy [22–24, 26].

### Deficiency of adenosine deaminase 2

DADA2, first described in 2014, is a rare, autosomal recessive, autoinflammatory, and immunodeficiency disease that manifests with features of a “PAN-like” vasculitis in the majority of cases [27•]. Many aspects of DADA2 remain enigmatic, and it is unclear whether the loss of the enzymatic activity in vitro is directly responsible for the disease manifestations. Many different pathogenic mutations in the ADA2 gene were described, and the phenotype is highly variable with features of vasculitis/vasculopathy, bone marrow disease (e.g., cytopenias), and immune dysregulation (e.g., hypogammaglobulinemia) [28, 29•]. DADA2 is a mimic of various disorders, including early stroke, connective tissue diseases, non-healing leg ulcers, hepatopathy, autoinflammatory diseases, and “PAN-like” MVV [30, 79]. Also, many patients with DADA2 fit under the diagnostic CVID umbrella [77•].

### Other PID

A single case of aortitis in an adult with hyper IgM syndrome (typically associated with susceptibility to bacterial infections) was identified [80]. In an adult with TAK and IBD, a gain-of-function germline mutation in STAT1 has been demonstrated [81]. STAT3-dependent hyperimmunoglobulin-E-syndrome (associated with significant IgE elevation, eczema, and eosinophilia) predisposes to aneurysm formation. Multiple cases with dilatations/aneurysms of the aorta, coronaries, carotids, or intracranial medium-sized arteries have been reported in adolescents and adults, some of which had histologically proven vasculitis [82]. DOCK8 deficiency, an autosomal recessive form of hyperimmunoglobulin E syndrome, typically manifests in childhood, but survival into adulthood is common and it can be associated with LVV (aortitis) or cerebral MVV [83].

## Malignancy and Paraneoplasia

All forms of vasculitis, but especially SVV (including AAV and IgA vasculitis) and MVV, have been observed in the context of neoplasia [39, 84]. However, a genuine paraneoplastic syndrome with synchronous disease progression of vasculitis and neoplasia is only rarely observed [85, 86]. Due to the relatively high incidence of malignancies and LVV in general, a purely coincidental occurrence of the two diseases is a likely scenario. In addition, patients with suspected vasculitis are often incidentally diagnosed with tumors during detailed imaging studies (such as FDG-PET-CT) [86]. With limited data overall, no specific solid tumor is associated with LVV and MVV [40, 85]. More relevant, however, is the association of vasculitides with hematologic neoplasms.

## Myeloid Neoplasms

### Myelodysplastic syndrome and myeloproliferative neoplasia (MDS, MPN)

10–25% of patients with MDS or chronic myelomonocytic leukemia (CMML) suffer from concomitant systemic inflammation with autoimmune or autoinflammatory manifestations, of which about one-third correspond to various types of vasculitis [31, 32, 36]. According to a large retrospective study, LVV was the most frequent subset, followed by "Behçet’s-like” syndrome, “PAN-like” vasculitis, and SVV (including AAV and cryoglobulinemic vasculitis). In approximately 44%, vasculitis is diagnosed prior to MDS/CMML and men seem to be affected more frequently. Vasculitis does not seem to have an impact on overall survival, and there is no specific association with MDS/CMML subtypes or their severity [34]. A recent case–control study described less prominent cranial symptoms in GCA in the context of MPN (including essential thrombocythemia, polycythemia vera, and CMML), especially with JAK2 mutations. In this context, corticosteroid dependence and a refractory disease course with shorter overall survival seem more common [33, 35]. *Acute myeloid leukemia (AML)/chronic myeloid leukemia (CML)*: MDS/MPN, including CMML, bear a high risk for progression to AML. Rarely, MVV or LVV can be observed in AML and CML. Apart from constitutional symptoms and symptoms related to cytopenias, leukemia can manifest with bone pain like a polymyalgic syndrome; examining the blood count in detail in is essential [31, 37–39, 41].

## Lymphoid Neoplasms

### Hodgkin’s and Non-Hodgkin’s lymphoma

Some studies describe an increased incidence of lymphoma (especially non-Hodgkin’s lymphoma) in patients with autoimmune diseases [87]. True paraneoplastic vasculitis is rare but was described in isolated cases, mostly affecting the medium-sized vessels (including the temporal artery in the form of GCA) and rarely the aorta and its major branches [37, 39, 42]. Interestingly, lymphoma (especially Hodgkin lymphoma) can be detected in up to 6% of patients with primary angiitis of the CNS [43, 44]. *Hairy cell leukemia (HCL)*: HCL is a rare lymphoid neoplasm which is occasionally associated with “PAN-like” vasculitis, and potentially affects the temporal arteries. Their simultaneous occurrence is almost certainly not coincidental, and “PAN-like” MVV should be considered a paraneoplastic phenomenon of HCL. Besides MVV, mainly cutaneous SVV can occur. Direct tumor cell infiltration of the vascular wall is also observed (a vasculitis mimic) [44–46].

## VEXAS (vacuoles, E1 enzyme, X-linked, autoinflammatory, somatic) syndrome

The VEXAS-Syndrome is a recently described disease due to somatic mutations in the *UBA1* gene on the X-chromosome (which explains why women are only exceptionally affected). It presents with a broad range of refractory inflammatory and hematologic manifestations such as polychondritis, cytopenias and macrocytosis, pulmonary infiltrates, and neutrophilic dermatosis. Various vasculitic manifestations can occur, ranging from cutaneous SVV/MVV (in 26% of patients) to temporal arteritis (in the form of GCA) or aortitis (in 1.7% of patients) [47••, 48]. Characteristic vacuolization in erythroid and myeloid precursor cells on bone marrow analysis is nearly always identified. VEXAS is associated with MDS in > 30% and is typically refractory to conventional immunosuppressive treatment strategies [88].

## Drug-Induced and Treatment-Related Vasculitis

Drug-associated vasculitis is common, and the list of triggering agents is long, including a broad range of pharmaceutical and illicit drugs such as cocaine (especially if cut with levamisole). However, SVV is far more common than LVV or MVV and commonly associated with serological abnormalities (elevated ANA, ANCA or antiphospholipid antibodies, low complement levels) [89, 90•].

### TNF-alpha inhibitors

Biologics, such as TNF-alpha inhibitors or rituximab, are indispensable therapeutics for immune-mediated diseases, but they can potentially trigger vasculitis, mainly SVV (especially cutaneous leukocytoclastic vasculitis or Henoch-Schonlein purpura) [91]. TNF-alpha inhibitors are reported to rarely induce MVV, including cerebral vasculitis or temporal arteritis, and LVV. The number of reported cases is small, and most of the affected patients suffered from rheumatoid arthritis. The clear differentiation between manifestations of the underlying rheumatoid arthritis with consecutive rheumatoid vasculitis and drug-induced vasculitis is difficult [91–93].

### Vaccines

The potential triggering of autoimmune diseases by vaccination is controversial. Various publications describe vasculitides of all forms in association with different vaccines [94]. In adults, GCA following influenza vaccination is the most discussed, and recent pharmacovigilance data revealed a potential safety signal for GCA and PMR with COVID-19 vaccinations [95, 96] There is a lack of studies demonstrating a clear association.

### Minocycline

This antibiotic is commonly used for long-term treatment in chronic acne in young patients. Its wide spectrum of side effects includes cutaneous and systemic MVV in the form of “PAN-like” vasculitis [49, 50].

### Immune checkpoint inhibitors (ICIs)

Immune therapy revolutionized cancer treatment and has become a well-established treatment modality for various neoplasms. ICIs are monoclonal antibodies that block regulatory immune checkpoints, such as programmed cell death protein 1 (PD-1) and others (PDL1 and CTLA-4), to enforce immunity against tumor-associated antigens [97]. Augmented anti-tumor immunity can be accompanied by the development of autoimmune phenomena, known as immune-related adverse events [97]. It is not surprising that the use of ICIs bears the risk of provoking vasculitis [98••, 99]. Although rare, LVV is the most common vasculitic complication of ICIs (esp. anti-PD1-therapy). It is responsive to the cessation of the ICIs and concomitant corticosteroid therapy. Fatalities attributable to vasculitis have not been reported [51, 52, 100•].

### Granulocyte-colony stimulating factor (G-CSF) and chemotherapy

G-CSFs, such as filgrastim, induce proliferation and maturation of neutrophils and are mainly used to treat chemotherapy-associated neutropenia. They bear the potential to induce a rapidly progressive LVV, with disease onset shortly after their first application. G-CSF withdrawal and corticosteroid treatment are effective with short recovery time. Certain chemotherapeutics (especially docetaxel) have been discussed as possible inductors of LVV [53, 54].

### Graft versus host disease (GVHD)

GVHD is a multiorgan disease due to donor cells initiating an immune response after engraftment in the host. GVHD rarely causes LVV or cerebral MVV [55, 56].

## Infectious Diseases

Infections are a rare but well-recognized etiology of vasculitis. Since inflammation of the vessel wall is observed, we consider infections to be the cause of true vasculitis. The underlying disease mechanisms include direct microbial invasion of the endothelial cells, immune-mediated injury of the vessel wall, stimulation of an immune response to shared epitopes between pathogens, and host or toxin-mediated injury [101, 102, 103•]. However, the available literature mostly consists of case reports or case series. Publications demonstrating a clear and unambiguous infectious etiology of vasculitides are scarce and limited to a few well-studied pathogens (e.g., hepatitis B virus (HBV); hepatitis C virus (HCV), *Salmonella* spp., and *Treponema pallidum* or *Mycobacterium tuberculosis*) [5]. Thus, the true incidence of infectious vasculitis remains unknown.

The enormous spectrum of microbes, the difficulty of pathogen detection from tissue samples, and the frequent necessity to diagnose infections based on serological tests (high risk for false-positive tests) make the unequivocal identification of a pathogen as the cause of vasculitis challenging. Serologic results and molecular detection of a pathogen must be reviewed critically and in the context of the clinical presentation, as testing can detect colonization or residual genetic material after resolved infection. It is often the patient’s medical history (including the social/professional and travel/migration history) that provides diagnostic clues and helps to narrow the spectrum of possible microorganisms and diagnostic workup. Table 2 and supplementary Table S2 summarize the most important microorganisms causing MVV and LVV from a Western European perspective.

### Bacterial infections

Staphylococci and streptococci are the most common *gram-positive* pathogens leading to MVV or LVV, usually of the aorta and the cerebral vessels [101, 102, 104, 105, 106•]. Rare reports describe aortitis or cerebral vasculitis caused by *Listeria* species (spp.) or *Mycoplasma* spp. [106•, 107–109]. Similarly, *Mycobacterium tuberculosis* is a known cause of aortitis as well as cerebral vasculitis [104, 106•, 110]. In the broad spectrum of *gram-negative bacteria*, *Salmonella* spp. (representing the Enterobacteriaceae) and *Treponema pallidum* (representing the Spirochaetaceae) are by far the most likely to cause vasculitis — usually aortitis [106•, 111–113, 115]. For *emerging bacteria* such as *Coxiella burnetii* and *Brucella* spp., *Francisella tularensis*, and *Leptospira* spp., cases with aortitis, cerebral or “PAN-like” vasculitis have been described [106•, 114, 116–121]. *Borrelia* spp. can cause cerebral MVV [106•]. While a single case of aortitis by *Tropheryma whipplei* was identified, clinically relevant MVV or LVV seem not to be significant manifestations [138].

### Viral infections: hepatitis B/C

Many different viruses are recognized as causative agents of vasculitis, but only HBV-associated vasculitis and HCV-associated cryoglobulinemic vasculitis are mentioned specifically in the Chapel Hill 2012 nomenclature [5]. HBV-associated “PAN-like” vasculitis is indeed one of the best studied forms of vasculitis; its prevalence and incidence have gradually declined thanks to extensive vaccination and screening programs [101, 105, 129, 130]. HCV is a frequent cause of cryoglobulinemic vasculitis, typically a SVV, but rarely manifesting in the form of a “PAN-like” vasculitis [3, 105]. *HIV*: Vasculitis only occurs in approximately 1% of HIV infections, and vessels of any size can be affected. The aorta and its large initial branches are mostly affected, but CNS vasculitis and “PAN-like” disease are also described [106•, 127, 128, 139]. *Herpesviridae*: Varicella zoster virus is most frequently associated with vasculitis among Herpesviridae; cerebral vasculitis is the typical manifestation [106•, 122]. A possible vasculitis of the temporal arteries has been debated for years, but a causative relationship has been found unlikely [140]. Occasional cases of herpes simplex virus with cerebral vasculitis have been described [123, 124]. Chronic active Epstein-Barr virus infection was linked to MVV or LVV (esp. aorta) in rare cases and more commonly in children; coronary aneurysms seem to be a typical complication [141, 142]. Information on Cytomegalovirus is scarce, but some well-described cases with “PAN-like” MVV were published [105, 125, 126]. *Parvovirus B19*: Although repeatedly described, it remains uncertain whether Parvovirus B19 truly causes LVV or MVV. “PAN-like” and CNS vasculitis were described mainly in children. A clear etiological link remains to be made. Since cryoglobulinemia and cold agglutinins (vasculitis mimic) can occur, these sequelae and their clinical manifestations (e.g., Raynaud’s phenomenon) need to be considered in patients with Parvovirus B19 [3, 101, 105, 106•, 143]. *SARS-CoV-2*: Early in the current pandemic, it became apparent that SARS-CoV-2 facilitates the induction of endotheliitis, contributing to thrombus formation in some cases with COVID-19. The multi-systemic inflammatory syndrome in adults can mimic Kawasaki disease, including coronary artery aneurysms, and it is clearly attributed to preceding/ongoing SARS-CoV-2 infection [144]. Further studies are needed to answer the question whether the few cases of vasculitis diagnosed in COVID-19 patients, ranging from SVV to LVV, are coincidental or are due to SARS-CoV-2 infection [103•, 145–148].

### Fungal infections

Vasculitides associated with invasive fungal infections are rare and occur almost exclusively in immunocompromised or critically ill patients or in special patient populations, such as intravenous drug users or migrants from endemic regions with dimorphic fungi. Not surprisingly, the related morbidity and mortality can be very high. *Candida* spp. is the most frequent fungus associated with vasculitis. Cerebral MVV is the most common vasculitis; however, large vessels like the distal carotids or aorta can be affected as well [105, 106•, 131–135].

### Parasites and infections in migrants and travelers

Depending on the patient’s migration or travel background and its related exposure risks, less frequently described pathogens like *Taenia solium* (mainly occurring in the southern hemisphere, causing Neurocysticercosis), *Toxocara* spp. or West Nile virus (occurring almost worldwide) need to be considered as rare causes of vasculitis, mainly of the cerebral vessels [136, 137, 149].

## Conclusion

Including a large variety of rare diseases and vasculitis mimics in the differential diagnosis of medium- to large-vessel vasculitis in adults is essential for correct diagnosis. This seems particularly important, if the diagnosis is made without histologic confirmation and is heavily based on imaging results. This review should be helpful in compiling a list of rarer and novel potential differential diagnoses in the appropriate clinical and epidemiological setting. Although the described disorders should be considered, they will undoubtedly remain rare in daily practice, as the primary vasculitides are much more common [3]. As new entities are constantly being described, we believe it is essential to periodically reevaluate atypical cases that have run for years under diagnoses that do not really fit the clinical phenotype. Also, we suggest performing a detailed diagnostic workup from the very beginning, because the accuracy of many diagnostic tests decreases quickly, once therapy is started.

Although we have attempted to be as comprehensive as possible, including very rare diseases, this was not possible for the section on infectious diseases. Given the enormous number of different microbes that could potentially cause vasculitis, we had to limit ourselves to the most commonly described germs. For tropical regions in particular, the scope of pathogens would have to be expanded considerably.

## Supplementary Information

Below is the link to the electronic supplementary material.

Supplementary file1 (PDF 161 KB)
