# Supplementary material for: Spectrum of Large- and Medium-Vessel Vasculitis in Adults: Neoplastic, Infectious, Drug-Induced, Autoinflammatory, and Primary Immunodeficiency Diseases
Source: Curr Rheumatol Rep. 2022 Aug 3;24(10):293–309. doi: 10.1007/s11926-022-01083-5 (PMC9362566; doi:10.1007/s11926-022-01083-5)
Supplement: Supplementary file 1 — Supplementary file1 (PDF 161 KB) [file 11926_2022_1083_MOESM1_ESM.pdf]

Supplementary Material for:

“Spectrum of Large- and Medium-Vessel Vasculitis in Adults: Neoplastic, Infectious, Drug-Induced, Autoinflammatory, and Primary Immunodeficiency Diseases”

Curr Rheumatol Rep ; https://doi.org/10.1007/s11926-022-01083-5

For references in Tables S1 and S2, please refer to the main text (the numbering of the references is the same like in the main text)

Table S1. Neoplastic, autoinflammatory and primary immunodeficiency diseases and drugs causing large and medium vessel vasculitis in adults

Category	Disease	Epidemiology, patient characteristics	Mainly affected large and medium vessels or vessel beds (i)		Clinical manifestations (ii)	Associated laboratory findings	Diagnostic pearls and pitfalls (iii)
			Large	Medium			
**Autoinflammatory Diseases**	**Familial Mediterranean Fever**	FMF: onset childhood to early adulthood, AR inheritance (MEFV gene), prevalence Mediterranean region higher. "PAN-like" MVV: ~ 1 % of FMF; m > f = 3.6:1; usually after onset of FMF. BS: associated with FMF in ~ 0.4 %, f >m [8,12,13]	+/- aorta (thoraco-abdominal); CCA, subclavian ("TAK-like") [10,11]	‡ "PAN-like" renal, cerebral, abdominal, cutaneous<br>+/- cardiac [8]	FMF: fever, abdominal pain (peritonitis), arthritis, rash. "PAN-like" MVV: constitutional symptoms and myalgia very common, hypertension, abdominal pain, perirenal hematoma, glomerular involvement (~ 35%), arthralgia/arthritis. BS-overlap: bipolar ulcers, skin/ocular/joints/CNS manifestation and pathergy. [8,13]	FMF: CRP/ESR ↑↑; serum amyloid A → / ↑; proteinuria (amyloidosis).<br>BS-overlap: ~ 50% HLA-B51 positivity. [8,9,13]	• Perirenal hematoma in ~ 50% (distinctive feature of "PAN-like" MVV)<br>• "BS-FMF-overlap": cutaneous, gastrointestinal and CNS involvement more frequent than in isolated BS [13]<br>• "PAN-like" MVV: compared to classical PAN, FMF patients are younger, testicular/cardiac involvement less frequent; CNS involvement more frequent and GN is possible.<br>• Hepatitis B Infection is detected in up to ~ 7% of "PAN-like" FMF [8]
	**Inflammatory Bowel Diseases**	IBD onset usually adolescence to early adulthood (onset at any age possible). IBD (esp. CD) and TAK or "TAK-like" at younger age (~ 20 Y/A) than isolated TAK. [23,24]	‡ "TAK-like" or TAK: aorta, subclavian, vertebral [23,24]	+/- "TAK-like" or TAK: renal, mesenteric, cerebral, cutaneous, TA (only with associated GCA) [23,24]	IBD: fever, weight loss, fatigue, abdominal pain, (bloody) diarrhea, extraintestinal symptoms: erythema nodosum, uveitis, arthritis, oral ulcers, CSVT. [27] (for manifestations of TAK and GCA see [3])	CRP/ESR → / ↑; fecal calprotectin ↑; atypical p-ANCA ↑ and anti-PR3-ANCA ↑ in CU, anti-Saccharomyces cerevisiae antibodies (ASCA) ↑ in CD [25,27]	• IBD preceding vasculitis in most cases in ~70%<br>• GCA with IBD rarely relapsing (in contrast to isolated GCA)<br>• IBD usually not active at time of vasculitis onset<br>• Due to limited data, differentiation of concomitant TAK or GCA and IBD vs. "TAK-like" and "GCA-like" disease with IBD not possible
	**Chronic Recurrent Multifocal Osteomyelitis**	Onset usually in childhood, but possible in adults; f > m; globally. LVV very rare (onset 3 to ~ 50 Y/A) [30,31]	‡ "TAK-like": aorta (ascending, descending), CCA, subclavian [30,31]	(iv)	Unifocal or multifocal sterile osteomyelitis (mandible, long bones of extremities, clavicles etc.) with associated local pain and occasionally soft tissue swelling. [30] (for manifestations of TAK and GCA see [3])	CRP and ESR ↑/↑↑ [30,31]	• Further associations with pyoderma gangrenosum, synovitis-acne-pustulosis-hyperostosis-osteitis syndrome (SAPHO) and IBD [30]
**Primary Immunodeficiency Diseases**	**Common variable immunodeficiency**	May manifest in childhood or in adulthood at any age; m = f; less common in developing nations; true prevalence of CVID unknown, estimated ~ 0.5-7 /10^6; any form of vasculitis in ~ 2% of CVID [33,35,36]	‡ "TAK-like" or TAK: aorta, innominate, CCA, ICA, axillary, subclavian [37,38,39]	+/- "TAK-like" or TAK: renal, mesenteric, celiac, coronary [37,38,39]	Frequent infections (respiratory > gastrointestinal > urinary > skin), fatigue, asthma, allergic rhinitis, atopy, urticaria, bronchiectasis, lymphadenopathy, splenomegaly, chronic diarrhea; (auto)-immune features (autoimmune cytopenias, thyreoiditis, IBD, granulomatous disease (sarcoid-like), vasculitis (purpura, large artery stenosis etc.), sicca syndrome, seronegative arthritis, hepatitis, gastritis, etc.). [36]	CRP → to mildly ↑; IgG ↓ (often <4.5g/L), IgG-subclasses →/↓, IgA and/or IgM ↓, IgE variable →/↓/↑; vaccine responses often ↓; lymphocyte flowcytometry (B-cells → > ↓ > ↑; B-cell subpopulations variable; T-cells and NK-cells usually normal); cytopenias possible, esp. platelets ↓ [35]	• Rule out other causes of hypogammaglobulinemia<br>• Detection of aortic aneurysm in CVID should trigger imaging for LVV<br>• Consider Deficiency of Adenosin-Deaminase-2 in CVID, esp. in patients with MVV (hypogammaglobulinemia is common in both)<br>• Screen for splenomegaly and lung disease in potential CVID<br>• Limited significance of any serologic test if patient is receiving immunoglobulin replacement therapy
	**Wiskott-Aldrich Syndrome**	Usually diagnosis in early childhood, exceptionally delayed to early adulthood in milder variants; X-linked recessive disorder; prevalence ~ 4/10^6; any form of vasculitis in ~ 1 – 29% [44,46]	‡ aorta (frequent aortic aneurysms, often panaortic);<br>+/- aortic arch arteries ("TAK-like") [48,49]	+/- cerebral, kidney, cardiac, liver, bowel, stomach [34,47]	Frequent infections (bacterial: respiratory, skin, enterocolitis, urinary, meningeal; viral: esp. herpesviruses), atopy, eczema, bleeding diathesis, autoimmune features in ~70% (cytopenias, arthritis, IBD, IgA nephropathy, vasculitis (purpura, aneurysms, large artery stenosis); secondary hematopoietic malignancies. [44-46]	Thrombocytopenia (20 - 70'000/µl) with small platelet volume; flowcytometry (T-cells and B-cells → /↓); IgG/M → /↓, IgA/E ↑; eosinophilia, neutrophils → /↓ (antibody mediated); vaccine responses ↓; multiple autoantibodies possible (incl. ANA subclasses) [44]	• With longer survival in WAS, aneurysms secondary to LVV may become more common; screening beginning in childhood might be justified<br>• Isolated presentation with thrombocytopenia is commonly called "X-linked thrombocytopenia", a mild variant of WAS<br>• Missing or reduced expression of the "WAS-protein" can be detected rapidly by lymphocyte flow cytometry in peripheral blood [44]
	**Deficiency of Adenosin-Deaminase-2**	Variable disease onset, can be delayed to adulthood; AR inheritance; m = f; globally (less common in Africa, East Asia); estimated prevalence ~ 4.5/10^6; any vasculitic feature ~ in > 75-90% [19,50,51]	(iv)	‡ "PAN-like" (including aneurysms): skin, muscle, mesenteric, celiac, hepatic, renal<br>+/- splenic, testicular, cerebral, coronary, pancreatic, TA [19,51,53]	Vasculopathy: livedo, skin ulcers, oral ulcers, digital necrosis, Raynaud's phenomenon, aneurysms of visceral arteries with bowel perforation, splenic/renal infarcts, testicular symptoms, neuropathy of peripheral and cranial nerves, ischemic (lacunar) > hemorrhagic stroke, etc. Other features: fever, spleno-/hepatomegaly, lymphadenopathy, arthralgia/arthritis, myalgia, hypertension, scleritis, mild susceptibility to respiratory tract and viral infections (e.g. warts). [19,51]	CRP mildly ↑ ~ 80%; any cytopenia ~ 50%; serum immunoglobulins (any type) ↓ ~ 20-65%; lymphocyte flowcytometry (abnormal ~ 80%, low class-switched memory B-cells ~ 70%, low memory T-cells/NK-cells ~ 50%); transaminases ↑; ANA/ANCA occasionally ↑; ADA2 enzymatic activity ↓↓ [19,51]	• Screening with ADA2 activity testing (e.g. with dried plasma spots)<br>• Biallelic mutations can be found in asymptomatic individuals (usually through screening of seemingly unaffected family members)<br>• Sneddon Syndrome is an important differential diagnosis (Livedo racemosa and CNS lesions)<br>• CNS involvement is much more common in DADA2 than in PAN<br>• Skin biopsy can show leukocytoclastic SVV and necrotizing MVV
**Malignancy - Paraneoplasia**<br>Myeloid Neoplasms	**Myelodysplastic Syndromes Myeloproliferative Neoplasms**	MDS/CMML: median onset ~ 70 - 75 Y/A (range 16 - 90 Y/A), m > f (MDS), m = f (CMML). MVV/LVV less frequent in polycythemia vera or essential thrombocythemia. [63,64,68]	+/- aorta, large veins ("BS-like" manifestation) [63,66,67]	‡ TA ("GCA-like")<br>+/- "PAN-like" (renal, hepatic, mesenteric, TA (non-GCA), cerebral, cutaneous) [63,65-67]	Constitutional symptoms, polymyalgic symptoms. Temporal arteritis in the context of MDS/CMML can present with the typical cranial symptoms of GCA, but they are less severe compared to idiopathic GCA. [63,65,66]<br>(For manifestations of BS, "PAN-like" MVV and GCA see [3])	ESR/CRP ↑/↑↑; eosinophils →/↑; cytopenias in MDS or MPN; monocytosis in CMML; thrombocytosis in essential thrombocythemia, erythrocytosis in polycythemia vera; myeloid somatic mutations incl. JAK2 mutations [65,68]	• Consider MDS/MPN in refractory MVV/LVV or with inflammatory dysimmune phenomena [65]<br>• The finding of cytopenia in LVV or MVV should lead to consideration of MDS or MPN as underlying disease process<br>• MDS with GCA has poorer outcome (relapses ↑, steroid dependency) [68]
	**Acute and Chronic Myeloid Leukemia**	Any age possible, incidence increases with age. AML: often progression of MDS/MPN. MVV/LVV very rare.	+/- "TAK-like": aorta, CCA, ICA, axillary, innominate, subclavian [69,71]	+/- "PAN-like": lower leg, TA ("GCA-like") [59,62,70,71]	Constitutional symptoms, livedo and ulceration of the skin, testicular pain, claudication and other manifestation of "TAK-like" disease. [70]	Leukemic findings in the blood count (leukocytosis, blasts); Philadelphia chromosome in Chronic Myeloid Leukemia.	• Basophilia and eosinophilia are common in Chronic Myeloid Leukemia.<br>• A differential blood count with visual inspection is advised in the setting of vasculitis with leukocytosis
Lymphoid Neoplasms	**Hodgkin and Non-Hodgkin Lymphomas**	Any age possible. Hodgkin and Non-Hodgkin lymphoma occasionally with LVV/MVV, multiple myeloma only rarely. [59]	+/- aorta, iliac, femoral [69]	‡ cerebral; +/- "PAN-like": renal, hepatic, mesenteric, infrabrachial, infrapopliteal, coronary; TA ("GCA-like") [59,69,73-75]	Constitutional symptoms, lymphadenopathy, hepatosplenomegaly. Skin ulcerations with MVV of distal arteries. CNS-symptoms (wide range). [73,74]	CRP/ESR ↑; eosinophils ↑ frequent; serum immunofixation potentially shows paraprotein; abnormal findings of cerebrospinal fluid (in cerebral MVV and/or cerebral lymphoma). [74]	• Lymphocyte flow cytometry of peripheral blood frequently shows monoclonality<br>• Intravascular lymphoma is a potential mimic of MVV, especially in the CNS or skin [3]
	**Hairy Cell Leukemia**	Mean onset ~ 50 Y/A (any age possible); m > f; MVV very rare. [75,76]	(iv)	‡ "PAN-like": cutaneous, hepatic, mesenteric, renal, cerebral, TA (occasionally TA aneurysm) [75-77]	Constitutional symptoms, splenomegaly, arthralgia, purpura (small vessel vasculitis), susceptibility to infections. [76]<br>Typical organ manifestation of classical PAN can occur (see [3]).	CRP/ESR ↑; pancytopenia, leukemic cells in bone marrow and peripheral blood smears [76]	• MVV is usually diagnosed in patients with known leukemia [75,76]<br>• "Hairy" cells can typically be identified in the peripheral blood smear
Other	**VEXAS-Syndrome**	Usually manifests ~ 50 - 80 Y/A; male >95%; associated MDS in > 30%; caused by somatic mutation in *UBA1*-gene; subset with polychondritis. [78,79]	+/- aorta [78,79]	‡ cutaneous (25%)<br>+/- TA [78,79]	Constitutional symptoms, skin lesions (neutrophilic dermatosis, leukocytoclastic vasculitis etc.), pulmonary infiltrates, serositis, polychondritis, ocular involvement, venous thromboembolism. [78]	CRP/ESR ↑ to ↑↑; cytopenias (all cell lines); macrocytosis; vacuoles in myeloid and erythroid precursor cells in bone marrow [78,79]	• Consider VEXAS in refractory cases with inflammatory dysimmune phenomena<br>• Elderly patient with autoinflammatory symptoms, cytopenias and/or polychondritis: look for vacuoles in bone marrow [78,79]
**Drug Induced Vasculitis**	**Minocycline**	Young patients (average 30 Y/A) with acne treatment (often long-term); f > m. [89,90]	(iv)	‡ "PAN-like": skin, nerves<br>+/- renal, mesenteric, gall bladder, liver, spleen, cervix [89,90]	Skin (subcutaneous nodules, livedo), hypertension, constitutional symptoms, peripheral nerves (paresthesia, rarely motor involvement). [89,90]	CRP and ESR often →; pANCA ↑ ~ 75% (occasionally MPO specificity); ANA ↑ ~ 50% [89,90]	• Onset of MVV on average ~ 26 months after initiation of minocycline therapy [89,90]
	**Immune Checkpoint Inhibitors**	Onset ~ 40 – 70 Y/A; f = m; vasculitis typically 1 – 3 months after initiation of treatment (*Ipilimumab, Pembrolizumab, Nivolumab*). [94,95]	+/- aorta [94]	‡ TA ("GCA-like"), cerebral (similar to primary angiitis of the CNS), uterine and ovarian vessels; peripheral nerves [94]	Immune related adverse events: pericarditis, myocarditis, gastrointestinal-symptoms, endocrine-metabolic disorders, hematological, ocular and many others; polymyalgic symptoms, arthritis. Typical organ manifestation of GCA can occur (see [3]). [94-96]	CRP/ESR ↑-↑↑, ANCA →/↑, ANA →/↑;<br>Depending on other immune related adverse events: cytopenias (including autoimmune hemolysis), hormone level disturbances, etc. [94]	• Vasculitis typically resolves after stopping immunotherapy (and/or a course of oral or intravenous glucocorticoids)<br>• No fatalities related to vasculitis observed [94]<br>• Overlap with other immunotherapy related adverse events possible (pericarditis, myocarditis, endocrine, gastrointestinal, etc.) [95]
	**Granulocyte-Colony Stimulating Factor**	Vasculitis occurs in ~ 0.3 - 0.5% of patients receiving G-CSF; mean ~ 60 Y/A; f > m [97,98]	‡ aorta (abdominal < thoracic, panaortic); carotids; +/- iliac, femoral, innominate, subclavian [97,98]	+/- TA [97]	Fever, malaise, pain (back, chest, abdominal and neck); "GCA-like" cranial symptoms including visual symptoms. [97,98]	CRP ↑ - ↑↑, preceding neutropenia (chemotherapy induced) [97,98]	• No fatalities related to vasculitis were observed<br>• In about 60%, vasculitis occurs within 10 days after G-CSF initiation (agents: Pegfilgrastim; Filgrastim; Lipefilgrastim; Lenograstim)
	**Graft-Versus-Host-Disease**	Vasculitis is a very rare manifestation	+/- aorta, iliac, femoral, subclavian, popliteal [100]	+/- cerebral [99]	Broad spectrum of clinical symptoms: skin rash, gastrointestinal (diarrhea, abdominal pain), liver dysfunction; non-focal and focal neurologic symptoms. [99,100]	CRP/ESR ↑↑; cerebrospinal fluid findings in CNS disease (pleocytosis, elevation of total protein etc.) [99]	• Cerebral vasculitis can manifest in long-term survivors [99]

Table legend Table S1:

- Notes: (i) Main vessels or vessel beds identified by our literature searches (i.e. arteries or vessel beds not listed, could still be affected). If not specified otherwise, the vessel names indicate arteries. The vessel sizes are defined as follows: “Large” (the aorta and distributing vessels of the extremities and neck, originating proximal to the elbow, knee and dura mater), “small” (arterioles, capillaries, venules and small intraparenchymal arteries and veins), “medium” (remaining vessels, including visceral arteries). (ii) A selection of important and pertinent clinical manifestations is provided; the list is not exhaustive, and the order is not according to frequency. (iii) Pearls mostly reflect the personal experience of the authors and are only partially referenced. (iv) Not identified by our literature search.
- Special characters: ‡ typically affected vessels or vessel beds; +/- occasionally to rarely affected vessels or vessel beds; ↑ elevated/more frequent/positive; ↑↑ markedly elevated; → no change/normal; ↓ depressed/below normal/less frequent; ~ approximately
- Abbreviations: ANA, antinuclear antibodies; ANCA, anti-neutrophil cytoplasmic antibody; AR, autosomal recessive; BS, Behçet’s Syndrome; CCA, common carotid artery; CD, Crohn’s disease; CNS, central nervous system; CMML, chronic myelomonocytic leukemia; CRP, C-reactive protein; CSVT, cerebral sinus vein thrombosis; CVID, common variable immunodeficiency; DADA2, Deficiency of Adenosine-Deaminase-2; ESR, erythrocyte sedimentation rate; FMF, Familial Mediterranean Fever; GCA, giant cell arteritis; G-CSF, Granulocyte-Colony Stimulating Factor; HLA, human leukocyte antigen; IBD, inflammatory bowel disease; ICA, internal carotid artery; Ig, immunoglobulin; LVV, large vessel vasculitis; MDS, myelodysplastic syndrome; MPN, myeloproliferative neoplasm; MVV, medium vessel vasculitis; PAN, polyarteritis nodosa; TA, temporal artery; TAK, Takayasu arteritis; UC, Ulcerative colitis; VEXAS, “vacuoles, E1 enzyme, X-linked, autoinflammatory, somatic”); WAS, Wiskott-Aldrich Syndrome; Y/A, years of age

Table S2. Infectious diseases causing large and medium vessel vasculitis in adults

Pathogen	Epidemiology, risk factors and patient characteristics	Mainly affected large and medium vessels or vessel beds (i)		Associated laboratory findings (ii)	Diagnostic pearls and pitfalls (iii)
		Large	Medium		
**Bacteria / Mycobacteria**					
**Gram-positive bacteria**					
**Gram-positive cocci (staphylococci, streptococci, enterococci)**	Any age; predisposing conditions for MVV/LVV: any infection with bacteremia (typically endocarditis), atherosclerosis, ICH, IVDA	‡ aorta, femoral, carotid (distal ICA) [101,102,104,105]	‡ cerebral: mainly anterior circulation, splanchnic. [101,105,106]	Positive cultures of blood or from tissue samples; positive PCR (specific or eubacterial) from vessel wall sample or blood; CSF with polynuclear pleocytosis and positive culture; urine: positive Streptococcal antigen	• Gram-positive bacteria most frequent microbiological etiology of any infectious vasculitis<br>• Vascular complications often first clue of infectious etiology (e.g. thrombosis, dissection/rupture)<br>• In suspected bacterial LVV/MVV, always look for local or distant occult infection, esp. endocarditis
***Listeria* spp.**	Elderly or young children; ICH; pregnancy; eating of contaminated products	‡ aorta (abdominal > thoracic) [107,108]	(iv)	Positive cultures of blood, tissue samples or CSF; positive PCR from tissue sample or blood	• Listeriosis more common in spring and summer season<br>• Screen for unknown immunodeficiency (e.g. hypogammaglobulinemia, HIV. etc.)
***Mycoplasma* spp.**	History of atypical pneumonia; exposure to infected children; in context of outbreak	(iv)	‡ cerebral [106,109]	Hepatitis common (ALT/AST ↑); in severe disease: cold agglutinins ↑; positive PCR from CSF and/or nasopharyngeal swab; positive serology (IgM/IgG)	• Disease and vasculitis rare in adults, more common in children; more common in summer and early fall<br>• Positive PCR or serology may be nonspecific due to high carriage rate; persistence of IgM and IgG possible for years
**Mycobacteria**					
M. tuberculosis	Travel (long-term) to or migration from high prevalence countries; close contacts (e.g. family member) with active pulmonary tuberculosis; ICH are at risk of reactivation of latent infection	‡ aorta (abdominal > thoracic); distal ICA [104,110]	+/- proximal cerebral arteries [106]	Anemia, thrombocytosis; ALT/AST ↑; tissue samples (vessel wall, lymph nodes, sputum, citrate blood) with visible acid-fast rods (Ziehl-Neelson stain); positive M. tuberculosis complex DNA PCR and culture; necrotizing granuloma in tissue samples	• Every organ can be affected; CNS involvement is rare, typically involves the basal intracranial structures<br>• Large samples and fluid volumes increase diagnostic yield; always check for HIV co-Infection<br>• Tuberculin skin test and interferon gamma release assay can only be used to rule out latent tuberculosis; confirmation of active infection requires positive samples of the affected organ
**Gram-negative bacteria**					
**Spirochetes**					
***Treponema pallidum* (Syphilis)**	Sexual activity; other sexually transmitted infections in the past	‡ ascending aorta<br>+/- aortic arch or descending aorta (rarely sinus or abdominal aorta) [112,113]	+/- cerebral: anterior circulation (esp. middle cerebral artery)<br>+/- TA [106,112,114]	Serum: positive TPPA (screening), positive RPR (confirmation). CSF: mixed or mononuclear pleocytosis, intrathecal TPPA antibody production and increased antibody CSF/serum index, positive RPR (confirmation). Histopathology: transmural inflammatory infiltration, typical involvement of vasa vasorum.	• CNS vasculitis can occur many years after the primary infection<br>• Imaging or surgical site typically shows thrombosed fusiform aortic aneurysms<br>• Always check for other sexual transmitted diseases and HIV co-Infection<br>• False positive TPPA/RPR test results can occur (e.g. HIV co-Infection and other infections)
***Borrelia* spp.**	History of tick bite or erythema migrans (< 50%); frequent outdoor activity in endemic regions	(iv)	‡ cerebral: SVV/MVV (diffuse and mainly leptomeningeal)<br>+/- dural sinus [106]	Serum: positive Borrelia immunoassay (screening), positive Borrelia immunoblot (confirmation); CSF: lymphocytic pleocytosis, intrathecal Borrelia antibody production and increased CSF/serum antibody index	• Neuroborreliosis occurs either in early disseminated infection, with intrathecal antibodies detectable after 2 weeks, or in late stage with intrathecal antibodies detectable in >99%<br>• Serologic testing should be performed with high clinical suspicion: serum antibodies and ↑ CSF/serum antibody index (without signs of inflammation in the CSF) may persist for years after resolved infection
***Leptospira* spp.**	Travel to warm and tropical regions, outdoor activity and animal contact (farmers, veterinarians, soldiers, canal workers (contact to contaminated soil and water) are at high risk)	‡ aorta [115]	+/- cerebral (size unclear), dural sinus; coronary [106,115]	Creatinine ↑; bilirubin ↑, ALT/AST ↑; positive blood, CSF and urine cultures (urine remains positive for 2-3 weeks); leptospira in dark field microscopy; positive serology (ELISA); positive microscopic agglutination test; positive PCR in serum, CSF and urine	• Rare disease, biphasic course with a flu-like early manifestation followed by convalescent phase<br>• CNS manifestations are often immune-mediated and present in late stages of the disease, thus CSF may be normal and culture negative<br>• Culture has a low sensitivity
**Other Gram-negative bacteria**					
***Salmonella* spp.**	Atherosclerosis, diabetes mellitus, ICH, hemoglobinopathies, abnormal intestinal mucosal barrier; eating of contaminated food	‡ aorta (abdominal > thoracic) [111]	(iv)	Positive blood and stool cultures, positive PCR (specific or eubacterial) from tissue sample	• Extra-intestinal manifestation in up to 40% of salmonella infections: bacteremia should prompt further search for infectious foci like aortitis, spondylodiscitis, osteomyelitis etc.
Coxiella burnetii	Travel to or migration from endemic regions; close contact to animals (e.g. cattle); occupational exposure (veterinarian, farmer); ICH; pregnancy	‡ aorta, mostly abdominal; +/- axillary [116,117]	+/- TA, "PAN-like" possible (hepatic) [117,118]	CRP ↑↑; AST/ALT ↑; positive serology, positive PCR and/or culture in tissue samples or blood; granuloma in tissue samples	• Preexisting aneurysm is a predisposing condition and must be distinguished from mycotic aneurysm resulting from septic emboli in Coxiella endocarditis.<br>• Primary infection is often asymptomatic, vascular involvement mainly in chronic infection
***Brucella* spp.**	Occupational exposure (farmer, animal breeder, butcher), travel to or migration from endemic regions, drinking of unpasteurized milk	‡ aorta (abdominal > thoracic), ICA [119,120]	+/- cerebral, dural sinus [120]	AST/ALT ↑; positive culture or PCR of blood, CSF or other tissue samples; positive serology in blood; CSF: mixed or neutrophilic pleocytosis, positive serology; granuloma in tissue samples	• False-positive serology possible due to cross-reactivity to other gram-negative bacteria<br>• IgG may persist lifelong, avidity of IgG helps to distinguish active disease and IgG-persistence<br>• Culture is less sensitive in chronic infection
Francisella tularensis	Outdoor activity or outdoor profession (e.g. forester, hunter), exposure to rodents, tick bites, contaminated material	‡ aorta (abdominal) [121]	(iv)	Positive serology; positive PCR from tissue samples; positive blood culture or less often from other samples; granuloma in tissue samples	• Disease spectrum ranges from mild symptoms (lymphadenitis) to pronounced constitutional symptoms
**Viruses**					
**Varicella Zoster virus**	Any age, ICH at risk	+/- ICA, vertebral arteries [106,128]	+/- cerebral [106,128]	CSF: predominantly lymphocytic pleocytosis, positive VZV DNA, intrathecal VZV-IgG production. Serum: usually VZV-IgG positive, IgM negative. Skin: positive VZV DNA of vesicle fluid. VZV antigen in tissue samples (vessel wall), Cowdry inclusion bodies in tissue samples.	• CNS manifestations can occur months after or even without typical VZV rash<br>• Intrathecal antibody production more reliable compared to PCR
**Herpes Simplex virus**	Any age; with or without comorbidities	+/- ICA [130]	‡ cerebral [130,131]	CSF: mononuclear (lymphocytic) pleocytosis, positive HSV-DNA, intrathecal HSV-IgG (later in disease course); serum: positive HSV serology	• Vasculitis is associated with HSV-2 (less frequently HSV-1)<br>• Absence of CSF pleocytosis (particularly in ICH) doesn't rule out HSV vasculitis, false negative PCR may occur in early illness; PCR should be repeated if clinical suspicion is high
**Cytomegalovirus**	Usually in heavily ICH	(iv)	‡ "PAN-like" (distal extremities, renal, splanchnic) [134,135]	Positive CMV PCR in blood and tissue samples; positive CMV serology (in primary infection)	• Primary infection typically occurs early in life (>70% of population seropositive) or after transplantation (donor positive organ to a CMV negative recipient)<br>• CMV reactivation in heavily ICH can affect any organ
**HIV**	Migrants from high burden countries, risk for STD, IVDA, mostly young or middle-aged patients	‡ aorta, carotid, subclavian, femoral, popliteal [126,127]	‡ cerebral<br>+/- "PAN-like" (skin, nerve, muscle, renal splanchnic) [106,126]	Lymphopenia (low CD4 T-cell); serum: reactive HIV-screening-test, positive confirmation assay (Western blot), positive p24-antigen, positive HIV-RNA; CSF: positive HIV-RNA	• Vasculitis is a very rare manifestation of HIV<br>• Main manifestation are aneurysms and occlusive diseases, independent of CD4 cell count and viral load. May be associated with immune reconstitution syndrome.<br>• Always test for other STDs and hepatitis B/C co-infection
**Hepatitis B virus**	Migrants from high burden countries (also second-generation: perinatal transmission), IVDA, sex workers	(iv) but highly likely	‡ "PAN-like" (skin, renal, testicular, splanchnic, peripheral nerves) [101,105,123,124]	AST/ALT: → to ↑↑; serology: positive HBs-antigen, usually negative anti-HBs-antibody, positive anti-HBc-antibody, positive or negative HBe-antigen, positive or negative anti-HBe-antibody; positive HBV-DNA (↑ to ↑↑); cryoglobulins not detectable to ↑	• Primary infection often subclinical and asymptomatic<br>• Hepatitis B associated cryoglobulinemic vasculitis possible<br>• Always check for Hepatitis C, D, and HIV co-infection
**Fungal infections (Yeasts (*Cryptococcus*, *Candida* spp.), Molds (*Aspergillus*, *Mucor* spp.), Dimorphic (*Histoplasma*, *Coccidioides* spp.))**	ICH, critically ill patients, IVDA; migration from and long-term travel to endemic regions (dimorphic fungi)	+/- aorta, carotids [144-146]	‡ proximal cerebral arteries (including mycotic aneurysms), esp. Candida and Aspergillus spp. [105,142,143]	Positive blood, CSF or tissue culture with yeasts, molds or dimorphic fungi; histological detection of fungi in tissue samples; Aspergillus: Galactomannan or Beta-D-Glucan; Candida: Beta-D-Glucan; Cryptococcus: Cryptococcus antigen in serum and CSF; Histoplasma: Histoplasma antigen in serum and PCR in tissue samples; Coccidioides: complement fixation IgG antibody test; all fungi: CSF occasionally with eosinophilic pleocytosis	• Candida is part of normal human flora, critical evaluation of positive cultures is indicated<br>• Sensitivity of Galactomannan is low and not standardized in serum and CSF<br>• High fatality rate in fungal vasculitis<br>• Screen for unknown immunodeficiency (e.g. hypogammaglobulinemia, HIV etc.)
**Parasites (v)**					
***Taenia Solium* (Neurocysticercosis)**	Migration from endemic region (seroprevalence in endemic regions is very high)	+/- ICA [148]	‡ cerebral ("cysticercal arteritis") [148]	Positive anti-cysticercal-antibodies; CSF: mild eosinophilic pleocytosis or normal, positive intrathecal anti-cysticercal-antibodies	• Symptoms can appear years after primary infection<br>• Cysticercal arteritis present in >6% of symptomatic neurocysticercosis patients
***Toxocara* spp.**	Close contact to dogs/cats (ownership); migration from and travel to endemic regions	(iv)	‡ cerebral (all vessels can be affected) [149]	Eosinophilia, serum: IgE ↑, positive serology, positive Western-blot; CSF: marked eosinophilic pleocytosis, positive intrathecal serology	• Serology cannot distinguish current and prior infection (cross-reactivity with other nematodes possible)<br>• Eosinophilia can persist over a year after treatment

Table legend Table S2:

- Notes: (i) Main vessels or vessel beds identified by our literature search (i.e. arteries or vessel beds not listed, could still be affected). If not specified otherwise, the vessel names indicate arteries. (ii) In infectious vasculitis, elevated C-reactive protein and/or leukocytosis is expected. (iii) Pearls reflect the personal experience of the authors. (iv) Not identified by our literature search. (v) Only two common parasitic infections are mentioned due to the rarity of such cases in Western Europe.
- Special characters: ‡ typically affected vessels or vessel beds; +/- occasionally to rarely affected vessels or vessel beds; ↑ elevated; ↑↑ markedly elevated; → no change/normal
- Abbreviations: ALT, alanine aminotransferase; AST, aspartate aminotransferase; CMV, Cytomegalovirus; CNS, central nervous system; CSF, cerebrospinal fluid; HSV, Herpes Simplex virus; ICA, internal carotid artery; ICH, immunocompromised host (e.g. immunosuppressive therapy, solid organ or hematopoietic stem cell transplantation, HIV); IVDA, intra-venous drug abuse; MVV, medium vessel vasculitis; LVV, large vessel vasculitis; PAN, polyarteritis nodosa; PCR, polymerase chain reaction; spp, species pluralis; STD, sexually transmitted diseases; SVV, small vessel vasculitis; VZV, Varicella Zoster virus
